# Role of PLP-Level as a predictive marker for oral health status in adult hypophosphatasia

**DOI:** 10.1007/s00784-024-05809-w

**Published:** 2024-07-08

**Authors:** Florian Dudde, Dominik Fildebrandt, Ralf Smeets, Martin Gosau, Michael Amling, Thomas Beikler, Florian Barvencik

**Affiliations:** 1 Department of Osteology and Biomechanics, University Medical Center Hamburg-Eppendorf, Lottestraße 59, 22529 Hamburg, Germany; 2 Department of Periodontics, Preventive and Restorative Dentistry, University Medical Center Hamburg-Eppendorf, Hamburg, Germany; 3 Department of Oral and Maxillofacial Surgery, University Medical Center Hamburg-Eppendorf, Hamburg, Germany; 4 Department of Oral and Maxillofacial Surgery, Division of Regenerative Orofacial Medicine, University Medical Center Hamburg-Eppendorf, Hamburg, Germany

**Keywords:** Adult, Hypophosphatasia, Oral health, PLP

## Abstract

**Aim:**

The aim of this study was to investigate the role of pyridoxal-5-phosphate (PLP) level on the oral health status as a predictive marker in patients with hypophosphatasia (HPP).

**Materials and methods:**

Throughout a systematic retrospective assessment both bone metabolism and oral health status were analyzed. The oral health status was assessed by the decayed/missing/filled teeth index (DMFT), clinical attachment level (CAL), probing pocket depth (PPD), and the periodontal screening index (PSI).

**Results:**

A total of 48 HPP patients (81.3% female) with a mean age of 42.21 years was included in this retrospective study. The study population was divided into two groups using the mean PLP level (87 µg/l) as a cut-off. Patients with a PLP level ≥ 87 µg/l (*n* = 14) showed a significantly poorer oral health status regarding DMFT index, CAL, PPD and PSI compared to patients with a PLP level < 87 µg/l (*n* = 34). No significant group differences for tooth loss were found.

**Conclusion:**

The results of the present study indicate that the PLP level is a suitable diagnostic predictor for the oral health status in HPP patients. HPP patients with PLP levels ≥ 70 µg/l should be included into a regular dental preventive program.

**Clinical Relevance:**

The oral health status in HPP and its correlation with laboratory parameters (i.e. PLP) has been understudied. For clinical practice, the findings of the present study clearly demonstrated that high PLP levels correlate with a worse oral health status in HPP patients. Therefore, these patients should receive an intensive dental treatment and/or inclusion in a strict maintenance program in a specialized dental practice/university hospital with a PLP level ≥ 70 µg/l.

## Introduction

Hypophosphatasia (HPP) is a rare metabolic disease with main manifestations in the mineralization process of the bone [1]. The prevalence of this disease varies between 1:508 and 1:300.000, depending on the subtype of HPP [2]. HPP is caused by pathogenic variants in the alkaline phosphatase liver/bone/kidney type (ALPL) gene, which codes for the non-tissue-specific alkaline phosphatase (TNSAP) [2]. This consecutively leads to reduced enzyme activity of TNSAP [3]. The impaired function of TNSAP causes a reduced cleavage of inorganic pyrophosphate (including pyridoxal-5-phosphate = PLP) into phosphate [2, 3]. The increased inorganic pyrophosphate levels directly impair the mineralization process while the lack of available phosphate additionally decreases bone mineral density [4]. HPP can be divided into different subforms based on the severity and the time of initial manifestation (perinatal, infantile, adolescent, adult, odontohypophosphatasia, pseudohypophosphatasia) [1, 2]. Adult HPP is one of the most common subforms of HPP [5]. However, the clinical symptoms of affected patients have a very wide spectrum [2, 6].

**Fig. 1:**
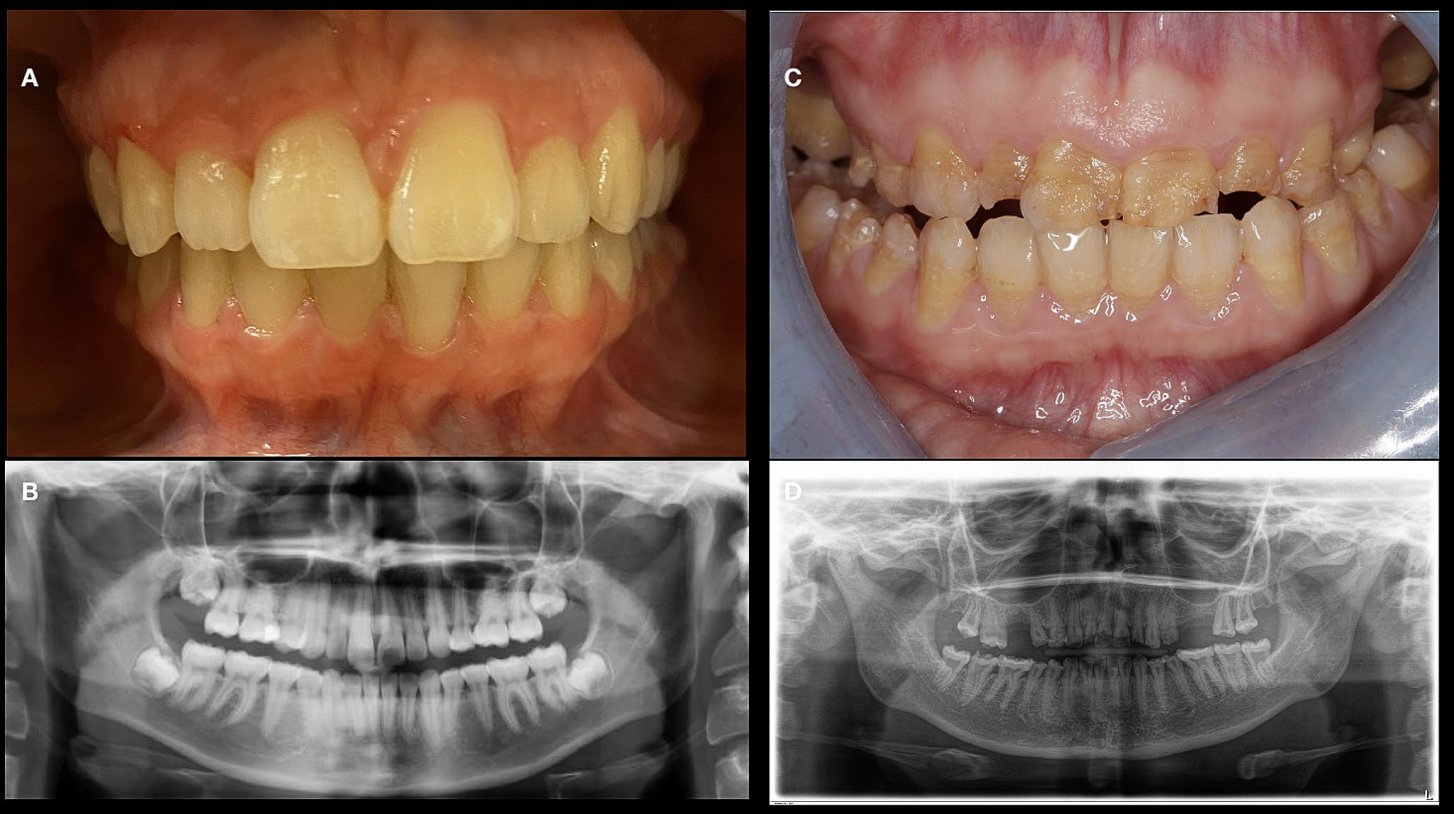
Clinical findings (Non-HPP patient vs. HPP patient). Direct comparison between Non-HPP patient (A, B) and HPP patient (C, D) based on clinical intraoral findings and associated panoramic radiograph

In addition to an increased susceptibility to fractures, myalgia, arthralgia, non-specific pain symptoms, and fatigue, many HPP patients, regardless of the subtype, often show a negatively affected oral health status [6, 7]. Previous studies have shown an increased number of atraumatic tooth loss of permanent teeth, early loss of primary teeth and an increased prevalence of periodontal disease in HPP patients [8–10]. This suggests that HPP affects mineralization not only in the bone, but also in teeth and adjacent structures (Fig. 1). Mineralization processes play a crucial role in the development and maintenance of dental hard tissue structures [11]. In this regard it has been shown that the mineralization disorders in HPP can have negative effects on the development of the alveolar ridge as well as the dental cementum [12]. Furthermore, hypoplastic forms of tooth enamel have been described in hard dental tissue structures in patients with HPP (Fig. 1) [13]. Anomalies in the dentin have also been found in HPP patients [14]. Anomalies and hypoplastic forms of teeth structures significantly increase the risk for the onset and progression of caries, ultimately leading to premature loss of primary and permanent teeth. [15].

In addition to that, Weider et al. showed a positive correlation between a negative oral health status of HPP patients and the number of mutations in the ALPL gene [10]. HPP patients (one/two ALPL mutations) had significantly higher decayed, missing and filled teeth (DMFT) indices compared to patients without HPP [10]. Weider et al. also demonstrated that patients with two mutations (compound heterozygous) in the ALPL gene had a significantly poorer periodontal status compared to patients without HPP [10].

This rare disease has been understudied in terms of oral health due to the limited number of individuals included in previous studies. Furthermore, no study on the correlation of PLP levels and the oral health status in HPP patients has been published yet. Therefore, the aim of this study was to investigate the role of the PLP level as a Predictive Marker for Oral Health Status in Adult Hypophosphatasia.

## Materials and methods

### Data collection

This retrospective study examined HPP patients who were treated in the Department of Osteology and Biomechanics at the University Hospital Hamburg and also in the Department of Periodontics, Preventive and Restorative Dentistry between 2017 and 2023. Only Patients with an adult form of HPP, that was confirmed through a genetic analysis (Pathogenic variants ALPL gene, ACMG class) accompanied by laboratory tests, were retrospective analyzed. All patients had a clear diagnosis of HPP based on the HPP major and minor criteria according to the International Working Group (IWG) on HPP [16]. The prerequisite for the diagnosis of HPP were the presence of 2 major criteria (pathogenic or likely pathogenic ALPL gene variant, elevation of natural substrates, atypical femoral fractures/pseudofractures, recurrent metatarsal fractures) or one major and two minor criteria (poorly healing fractures, chronic musculoskeletal pain, early atraumatic loss of teeth, chondrocalcinosis, nephrocalcinosis) [16]. The patients were fully capable of consenting to the study. Exclusion criteria were incomplete documentation (patient records). Data collection regarding bone metabolism and clinical findings was carried out using the patient files in the Department of Osteology and Biomechanics. The data collection of the oral health status was carried out from the patients’ digital patient files (Charly Program by Solutio, Holzgerlingen, Germany) in the Department of Periodontics, Preventive and Restorative Dentistry. A total of 48 patients were included in this study. All patients provided written informed consent before participating in any study within the Department of Osteology and Biomechanics.

### Clinical data

For each patient, the clinical baseline characteristics (age, gender, body-mass-index = BMI, mutation, ACMG class) as well as laboratory parameters (alkaline phosphatase = AP, bone specific alkaline phosphatase = bAP, PLP, Vitamin D, Magnesium, Copper, Phosphate, Calcium, parathyroid hormone = PTH) were analyzed. Furthermore, the results of the dual x-ray absorptiometry (DXA) scan were also analyzed for these patients. DXA is the most common used method for measuring osteodensitometry. Using DXA, measurements of bone density a can be determined preferably on the spine, femur (femoral neck) and other specific regions [17]. The values of DXA are presented in T-Scores and Z-Scores. The T-score is determined in order to decide when measuring bone density whether osteoporosis is present or not. It is presented in standard deviations (SD) referring to the bone density of healthy people aged 30 [18]. In addition to the T-score, the Z-score is another bone density measurement parameter that can provide an indication of whether osteoporosis needs to be treated or not. The Z-score represents the standard deviation of the measured bone density from the mean of a comparison group of the same age [18]. The intraoral examination was carried out within two weeks after initial diagnosis of HPP and simultaneous serum collection.

### Oral health status

During the routine dental examination the following parameters were surveyed:

1. Decayed missing filled/tooth (DMFT) index.
2. Number of natural teeth in situ (excluding third molars).
3. Periodontitis screening index.
4. Periodontitis severity grade (PSI).
5. Probing Pocket Depth (PPD).
6. Clinical attachment level (CAL).

The PPD and CAL were determined using a standardized manual periodontal probe (CP-11; Hu-Friedy, USA). Following the study by Holfreter et al. PPD and CAL were recorded on six sides of the tooth (mesiobuccal, buccal, distobuccal, mesiooral, oral and distooral) [19]. The patients were also asked at what age the first permanent tooth erupted. The oral assessment was performed by at least two years trained, experienced dentists, in the field of periodontology (DF, TB).

### PLP-Level distribution

The present study aimed to determine the correlation of PLP levels with the oral health status in HPP patients. Consequently, based on the mean PLP level (cut-off 87 µg/l), the study population was divided into two groups (group A with a PLP level ≥ 87 (µg/l) (*n* = 14) vs. groups B with a PLP level < 87 (µg/l)).

### Statistical analysis

Descriptive analysis was used to display patients baseline characteristics. Normally distributed continuous variables are presented as mean ± standard deviation and binary variables are using absolute and relative frequencies. Comparison of continuous variables was performed by student’s t-test. Chi- square test was used for analysis of binary variables. Scatterplots including trend line analysis were used as graphic elements. A p-value < 0.05 was considered statistically significant. All statistical analyses were performed using the SPSS version 28.0 statistical package (IBM, Markham, Canada).

## Results

### Baseline

A total of 48 patients with genetically confirmed diagnosis of HPP was included in the present study. The data for each patient on bone metabolism and dental status were collected as outlined in the Material and Methods section. The average age of all patients for the entire study population was 42.21 years (Table 1). A total of 9 (18.8%) men and 39 (81.3%) women were included in the study (Table 1). The average BMI was 24.35 kg/m² (Table 1). Furthermore, the respective HPP variant was analyzed according to the ACMG classification (Table 1) [20]. For the entire cohort, in descending relative frequency, 50.0% mutations were ACMG class 5, 33.3% mutations were ACMG class 3 and 16.7% mutations were ACMG class 4 respectively (Table 1). Based on the PLP level (mean 87.10 µg/l), the study participants were divided into two groups (Table 1). The study was divided into group A with a PLP level ≥ 87 (µg/l) (*n* = 14) and group B with a PLP level < 87 (µg/l) (*n* = 34) (p = < 0.001) (Table 1). The alkaline phosphatase level was 33.83 U/l for the entire cohort, 26.86 U/l for group A and 36.71 for group B (Table 1). Furthermore, bone-specific alkaline phosphatase was reduced to 3.97 U/l for patients with a PLP ≥ 87 µg/l compared to patients with a PLP < 87 µg/l and a value of 6.74 U/l (Table 1).

No significant differences between the two groups were found regarding the magnesium level, the copper level, the phosphate level, the parathyroid hormone level nor the vitamin D level (Table 1). The calcium level in group A was 2.43 mmol/l compared to group B with a value of 2.34 mmol/l (*p* = 0.03) (Table 1).

**Table 1:** Baseline characteristics (*n* = 48)

Variable	Total (*n* = 48)	PLP ≥ 87 µg/l (*n* = 14)	PLP < 87 µg/l (*n* = 34)	*p*-Value
Age	42.21 (± 15.78)	46.79 (± 12.69)	40.32 (± 16.70)	0.201
Gender				0.760
Male	9 (18.8)	3 (21.4)	6 (17.6)	
Female	39 (81.3)	11 (78.6)	28 (82.4)	
BMI (kg/m²)	24.35 (± 5.86)	25.52 (± 5.50)	23.87 (± 6.02)	0.382
ACMG Class				0.867
1	0 (0)	0 (0)	0 (0)	
2	0 (0)	0 (0)	0(0)	
3	16 (33.3)	4 (28.6)	12 (35.3)	
4	8 (16.7)	3 (21.4)	5 (14.7)	
5	24 (50.0)	7 (50.0)	17 (50.0)	
PLP (µg/l)	87.10 (± 68.31)	148.84 (± 86.91)	47.14 (± 18.88)	**< 0.001**
AP (U/l)	33.83 (± 20.83)	26.86 (± 9.80)	36.71 (± 23.47)	0.138
bAP (U/l)	5.93 (± 7.23)	3.97 (± 2.19)	6.74 (± 8.38)	0.232
Magnesium (mmol/l)	0.85 (± 0.07)	0.86 (± 0.07)	0.85 (± 0.07)	0.473
Copper (µg/l)	1075.55 (± 395.43)	1072.55 (± 222.04)	1076.78 (± 451.17)	0.977
Calcium (mmol/l)	2.37 (± 0.12)	2.43 (± 0.14)	2.34 (± 0.11)	**0.030**
Phosphate (mmol/l)	1.14 (± 0.26)	1.24 (± 0.25)	1.10 (± 0.25)	0.084
Vitamin D (µg/l)	28.98 (± 15.39)	23.98 (± 10.63)	31.04 (± 16.66)	0.151
PTH (ng/l)	44.15 (± 18.82)	42.01 (± 15.21)	45.03 (± 20.26)	0.620

Note: *Data are presented as mean ± SD and/or percentage*

Abbreviations: AP = alkaline phosphatase, bAP = bone specific alkaline phosphatase, BMI = Body-mass-index; ACMG = American College of Medical Genetics, PLP = Pyridoxal phosphate, PTH = Parathyroid hormone.

### DXA-Scan

In the present study, patients with a PLP ≥ 87 µg/l showed higher values ​​for bone mineral density for the spine, left femur and right femur compared to patients with a PLP < 87 µg/l (Table 2). Consequently, lower values ​​for the Z-scores and T-scores of the right and left femur were found in patients with a PLP < 87 µg/l (Table 2).

**Table 2:** DXA scan (SD)

Variable	Total (*n* = 48)	PLP ≥ 87 (µg/l) (*n* = 14)	PLP < 87 (µg/l) (*n* = 34)	*p*-Value
BMD SC (g/cm²)	1.14 (± 0.22)	1.20 (± 0.21)	1.12 (± 0.22)	0.140
T-Score SC	− 0.16 (± 1.59)	0.09 (± 1.74)	− 0,29 (± 1.53)	0.489
Z-Score SC	− 0.03 (± 1.33)	0.37 (± 1.36)	− 0,21 (± 1.30)	0.176
BMD Left Femur (g/cm²)	0.95 (± 0.15)	0.97 (± 0.15)	0.94 (± 0.15)	0.481
T-Score Left Femur	− 0.49 (± 1.10)	− 0.45 (± 1.03)	− 0,50 (± 1.16)	0.880
Z-Score Left Femur	− 0.21 (± 0.98)	− 0,11 (± 0,91)	− 0,25 (± 1.02)	0.671
BMD Right Femur (g/cm²)	0.94 (± 0.14)	0.97 (± 0.15)	0.93 (± 0.14)	0.353
T-Score Right Femur	− 0.57 (± 1.00)	− 0.47 (± 1.00)	− 0.62 (± 1.02)	0.666
Z-Score Right Femur	− 0.28 (± 0.95)	− 0,13 (± 0.90)	− 0,34 (± 0.98)	0.492

Note: *Data are presented as mean ± SD*

SC = Spinal column, BMD = Bone mineral density.

### Oral health status – DMFT

**Fig. 2:**
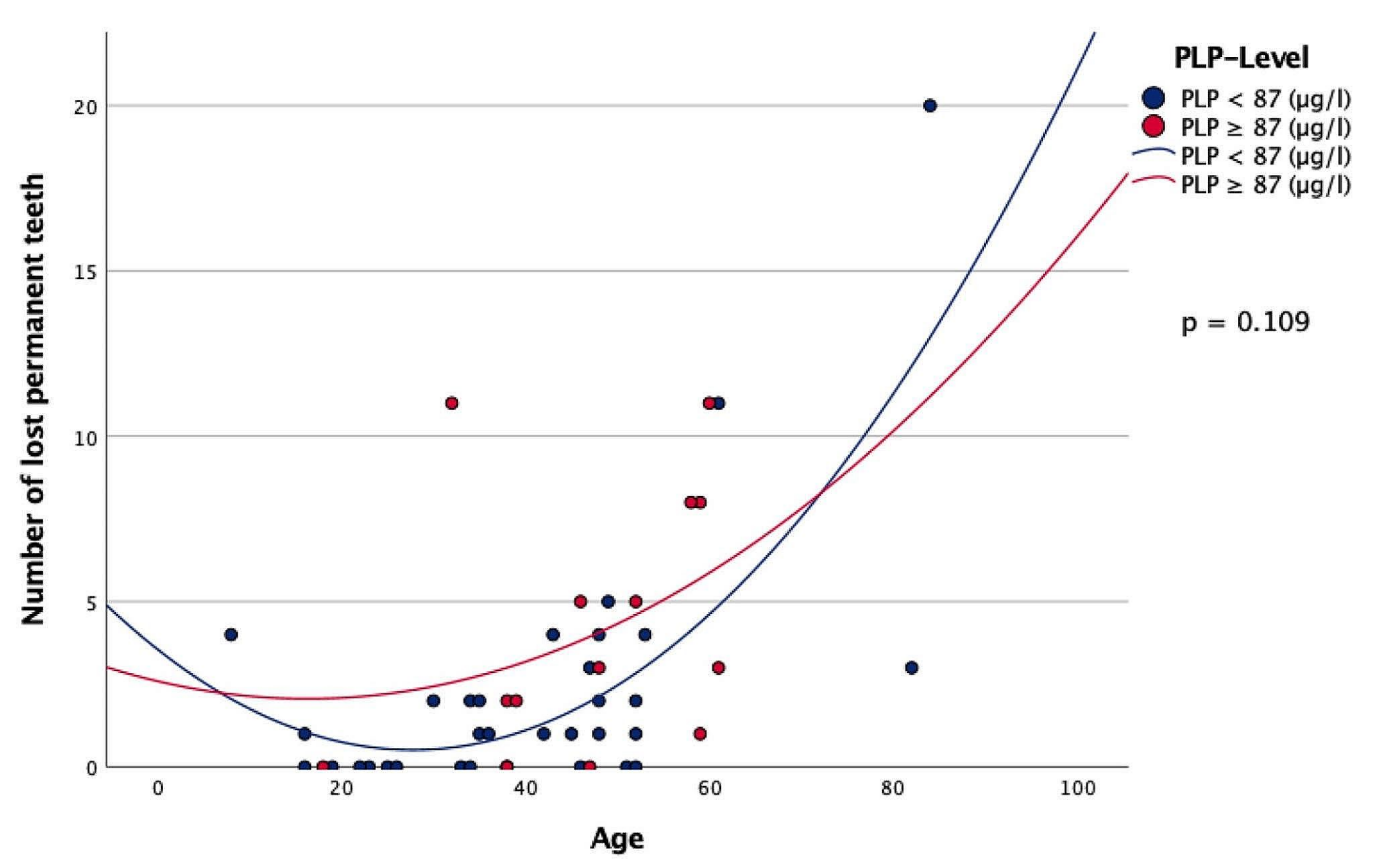
Scatterplot - Number of lost permanent teeth

**Fig. 3:**
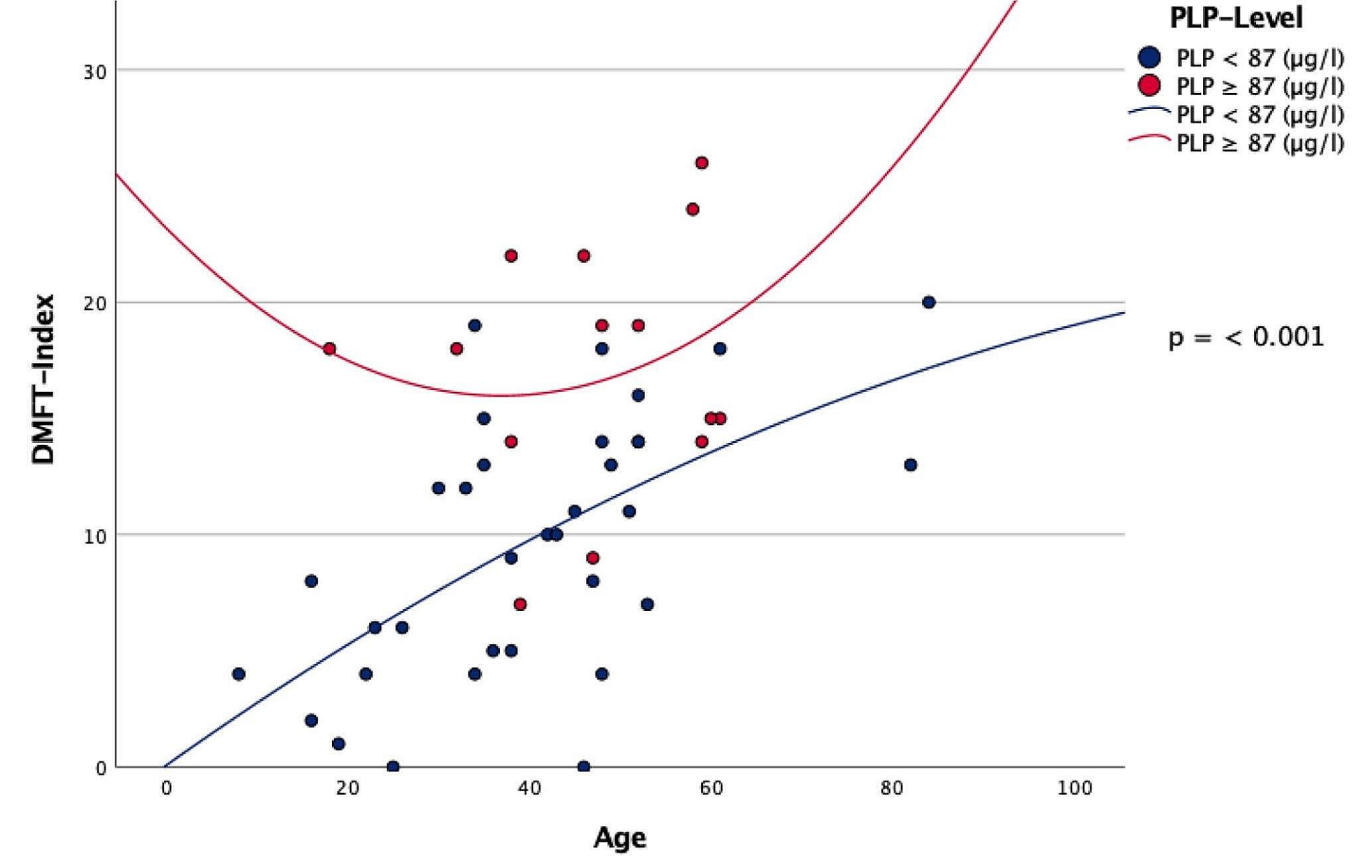
Scatterplot – DMFT-Index

When comparing both cohorts, patients with a PLP ≥ 87 µg/l had lower numbers of natural permanent teeth in situ (23.79 teeth) compared to patients with a PLP < 87 µg/l and a total number of 25.79 teeth (Table 3). Furthermore, patients in group A showed a higher number of decayed teeth (2.86) compared to group B (1.85 decayed teeth) (Table 3). Patients with a PLP ≥ 87 µg/l consequently showed a higher prevalence of missing teeth (4.21) compared to patients with a PLP < 87 µg/l and a total of 2.21 missing teeth (Table 3; Fig. 2). There were highly significant differences between the two groups regarding the number of filled teeth (Table 3). The DMFT Index was significantly increased in patients with PLP ≥ 87 µg/l (17.29 DMFT-index) compared to patients with PLP < 87 µg/l and a DMFT-index of 9.59 (Table 3; Fig. 3).

**Table 3:** Oral health status – DMFT in HPP patients (SD)

Variable	Total (*n* = 48)	PLP ≥ 87 (µg/l) (*n* = 14)	PLP < 87 (µg/l) (*n* = 34)	*p*-Value
Number of natural permanent teeth	25.21 (± 3.94)	23.79 (± 3.91)	25.79 (± 3.85)	0.109
Decayed	2.15 (± 2.44)	2.86 (± 2.98)	1.85 (± 2.16)	0.198
Missing	2.79 (± 3.94)	4.21 (± 3.91)	2.21 (± 3.85)	0.109
Filled	6.90 (± 4.76)	10.21 (± 4.39)	5.53 (± 4.25)	**< 0.001**
DMFT-Index	11.83 (± 6.53)	17.29 (± 5.40)	9.59 (± 5.61)	**< 0.001**

Note: *Data are presented as mean ± SD*.

### Oral health status – periodontal status

Regarding the periodontal health, there were significant differences between the two groups focusing on the PSI (Table 4). Patients with a PLP ≥ 87 µg/l showed significantly higher PSI values ​​than patients with a PLP < 87 µg/l (Table 4). There were also significant differences between the two groups based on the severity classification of periodontitis (Table 4) [21]. Patients with a PLP ≥ 87 µg/l showed significantly higher relative frequencies of severe periodontitis (35.7%) compared to patients with a PLP < 87 µg/l (5.8%) (*p* = 0.029) (Table 4).

**Table 4:** Detailed Periodontal data (SD and/or percentage) in HPP patients according to Holtfreter et al. [19]

Variable	Total (*n* = 48)	PLP ≥ 87 (µg/l) (*n* = 14)	PLP < 87 (µg/l) (*n* = 34)	*p*-Value
PSI - S1	2.19 (± 1.02)	2.71 (± 0.91)	1.97 (± 1.00)	**0.021**
PSI - S2	1.94 (± 0.93)	2.64 (± 0.84)	1.65 (± 0.81)	**< 0.001**
PSI - S3	2.13 (± 1.04)	2.71 (± 0.91)	1.88 (± 1.01)	**0.011**
PSI - S4	2.04 (± 0.97)	2.64 (± 1.01)	1.79 (± 0.85)	**0.004**
PSI - S5	2.10 (± 1.03)	2.64 (± 0.93)	1.88 (± 0.64)	**0.002**
PSI - S6	1.92 (± 1.03)	2.71 (± 0.91)	1.59 (± 0.89)	**< 0.001**
Sites per Mouth CAL > 3 mm (%)	11.80 (± 12.62)	19.89 (± 15.87)	8.48 (± 9.41)	**0.003**
Sites per Mouth CAL > 5 mm (%)	2.31 (± 6.10)	5.49 (± 8.96)	1.00 (± 3.92)	**0.019**
Teeth per Mouth CAL > 3 mm (%)	13.10 (± 12.09)	20.93 (± 14.77)	9.87 (± 9.25)	**0.003**
Teeth per Mouth CAL > 5 mm (%)	2.38 (± 5.41)	5.71 (± 8.11)	1.01 (± 3.03)	**0.005**
Sites per Mouth PPD > 4 mm (%)	11.55 (± 19.11)	24.69 (± 28.92)	6.14 (± 9.34)	**0.001**
Sites per Mouth PPD > 6 mm (%)	3.69 (± 9.70)	8.24 (± 12.62)	1.82 (± 7.67)	**0.036**
Teeth per Mouth PPD > 4 mm (%)	20.30 (± 24.09)	34.36 (± 30.06)	14.51 (± 18.77)	**0.008**
Teeth per Mouth PPD > 6 mm (%)	4.29 (± 11.36)	10.52 (± 18.57)	1.73 (± 4.97)	**0.013**
Age at first tooth exfoliation	5.06 (± 0.63)	5.14 (± 0.66)	5.03 (± 0.63)	0.578
Periodontitis Severity Grade*				**0.029**
No/mild PD	27 (56.3)	6 (42.9)	21 (61.8)	
Moderate PD	14 (29.2)	3 (21.4)	11 (32.4)	
Severe PD	7 (14.6)	5 (35.7)	2 (5.8)	

Note: *Data are presented as mean ± SD and/or percentage*

Abbreviations: PSI = Periodontitis screening index, CAL = Clinical attachment level, PPD = Probing pocket depth, PD = Periodontitis, * Eke PI, et al. Update of the case definitions for population-based surveillance of periodontitis. J Periodontol. 2012 [21]

In addition, group A showed significantly higher relative frequencies of sites with a CAL > 3 mm / 5 mm in comparison to group B (Table 4). The number of sites with a PPD > 4 mm / 6 mm was also significantly increased in patients with a PLP ≥ 87 µg/l (Table 4). These significant differences could also be detected for the frequencies of teeth with a CAL > 3 mm/5 mm as well as PPD > 4 mm/6 mm (Table 4).

## Discussion

HPP is considered a rare disease with a variety of clinical symptoms. A manifestation in the oral health status of this systemic disease can be observed in almost every type of HPP [6, 7]. It is important to note that there is a scarcity of studies focusing on the oral health status of HPP patients. The recent publication by Weider et al. provides the only available systematic examination of the oral health status in HPP patients [10].

Regarding population size, age and gender distribution, a direct comparison with the study by Weider et al. showed similar study conditions [10]. One of the most essential parameters when determining the oral health status is the widely studied and standardized DMFT index [22]. Regarding the DMFT index in HPP patients, the present study showed significantly higher DMFT indices in patients with a high PLP level correlating with a poorer oral health status. In direct comparison with the before mentioned publication, the HPP patients of the present study showed lower DMFT values (17.0 vs. 11.83) [10].

However, in the study by Weider et al. the number/variants of TNSAP mutations correlated with a higher DMFT index (22.4), which is consequently associated with lower enzyme activity and higher PLP levels and is therefore similar to the DMFT index results of the present study (PLP ≥ 87 µg/l = DMFT 17.29) [10]. These results can also be reproduced in comparable values ​​on filled teeth in patients with a PLP ≥ 87 µg/l in the present study and patients with two TNSAP variants (24–45 years) (10.21 vs. 9.8) [10].

Furthermore, a direct comparison with the results of the *Deutsche Mundgesundheits-studie V* (DMS V) study also showed higher DMFT indices in HPP patients compared to the age-related German reference cohort (DMFT Index 35–44 years = 11.2) [23, 24]. However, clear differences could be demonstrated regarding the number of “decayed” teeth. For the entire HPP cohort of the present study, there was a mean decayed teeth value of 2.15, respectively 2.85 for patients with higher PLP values. The number of decayed teeth in the study by Weider et al. was only 0.9 (one variant) and 1 (two variants) respectively [10]. The results of the present study on decayed teeth continue to significantly exceed the number of decayed teeth in the DMS V study and thus indicate a poorer oral health status in HPP patients compared to the German reference population [23, 24]. Therefore, these findings confirm the assumed increased prevalence of decayed teeth in HPP patients [3, 25].

Another parameter that remains essential for the oral health status in HPP patients is the periodontal health assessment in accordance with CDC/AAP classification [10, 19]. In the present study, patients with a PLP ≥ 87 µg/l showed significantly higher levels of periodontitis severity than patients with a PLP < 87 µg/l. In addition to that, a comparable study proved that the number of variants of TNSAP was associated with a significantly increased prevalence of moderate to severe periodontitis, which underlines the finding of the present study [10]. This can also be demonstrated in comparable higher values ​​of PPD and CAL compared to the DMS V German reference cohort [23, 24].

Patients with a PLP ≥ 87 µg/l also showed significantly higher PSI values, thus confirming the clinical extent of periodontitis. Furthermore, HPP patients showed significantly increased values ​​on teeth with a higher CAL and PPD correlating with a higher risk/degree for severe periodontitis. In comparison, the present study showed significantly higher prevalence of teeth with a PPD > 4 mm / > 6 mm and conversely lower values ​​of teeth with a CAL > 3 mm / 5 mm [10]. Further studies have already shown that HPP patients tend to have an increased risk of periodontitis [26]. The biological plausibility of this observation can be demonstrated with the help of preliminary work [27]. The increased PLP levels result from a severely restricted enzymatic activity of TNSALP. Since TNSALP is also found in ameloblasts, cementoblasts, odontoblasts and the periodontal ligaments (PDL), the mineralization disorders are also consistent in the oral cavity [27]. Wölfel et al. examined teeth from two patients with infantile HPP using quantitative backscattered electron imaging (qBEI) and histological analysis [27]. In HPP teeth, the calcium content of the cementum was significantly lower in the HPP patient compared to healthy controls [27]. Furthermore, HPP teeth showed severe hypomineralizations in the dentin in comparison to the control group [27]. Interestingly, differences regarding the absence of acellular cementum were demonstrated in their study as well [27]. While the absence of acellular cementum in control teeth was due to root resorption, in HPP patients acellular cementum was absent even though the roots were existing consequently leading to a higher repair cementum thickness in HPP teeth [27]. Summarizing, the lower enzyme activity of TNSALP with increased PLP values consequently increases the absence of acellular cementum, the hypomineralization of the cementum and negatively affects the periodontal ligament [27]. Therefore, a significantly worse periodontal status can consequently be assumed, as it was proven in the present study (Table 4) [27, 28].

However, the extent or clinical presentation of these parameters in HPP does not seem to be fully understood. Since the periodontal health assessment (PPD, CAL) is essential regarding the preservation of permanent teeth, there is a need for further examinations in order to avoid early tooth loss in HPP patients and to identify other risk factors/patterns in HPP patients.

The present study showed that there are no significant differences in the age of primary tooth loss based on PLP level. Notably, unlike other publications, these findings did not show higher rates of early primary tooth loss or younger age of first permanent tooth exfoliation [8, 24, 29].

In the present study no significant differences in the DXA measurements (T-score, Z-score) with regard to the PLP level were observed. Comparable studies were also unable to demonstrate a clear association of the PLP level with all DXA findings [30, 31]. Genest et al. showed a negative correlation of the ALP level (consecutively higher PLP level) with the lumbar spine T-score according [30]. However, in their study the ALP level (consecutively the PLP level) did not correlate significantly with the femoral T-scores [30]. Therefore, a correlation of ALP/PLP level with DXA findings remains unclear to this point even though these biochemical markers are associated with an increased disease severity in HPP [31]. In addition to that Schmidt et al. also could not demonstrate a correlation of ALP/PLP levels with higher T-scores/Z-scores in their study of 38 patients with adult HPP [31]. A possible explanation may be the comparatively small study groups since this observation is not fully understood. Consequently, there is a need for further studies to investigate the influence of the PLP level and/or other biochemical parameters on the DXA findings in adult HPP.

The results of this study are limited due to several facts. The present study is a monocentric work with a limited population size. Furthermore, the oral health status data of the participants was retrospectively analyzed by only two examiners without any follow-up intervals, such as after enzyme replacement therapy or accompanying dental procedures.

However, the aim of this study was to investigate the correlation of the PLP level with the oral health status as a diagnostic marker in HPP patients. In the present study, patients with a PLP level ≥ 87 µg/l showed a significantly worse oral health status (DMFT, periodontal health status) than patients with a PLP level < 87 µg/l. Consequently, taking the aforementioned limitation into account, it can be concluded that the PLP level is suitable as a diagnostic predictor for the oral health status in HPP patients. For clinical practice, it can also be concluded that HPP patients should receive an intensive dental treatment and/or inclusion in a strict maintenance program in a specialized dental practice/university hospital with a PLP level ≥ 70 µg/l. The results of the study clearly demonstrate significant differences in the oral health status based on the PLP level with a cut-off of PLP ≥ 87 µg/l. Further multicenter studies with larger HPP populations need to be conducted in order to confirm these results and establish PLP values as a reliable predictor for oral health status in HPP patients.

## Data Availability

No datasets were generated or analysed during the current study.
